# BRGenomics for analyzing high-resolution genomics data in R

**DOI:** 10.1093/bioinformatics/btad331

**Published:** 2023-05-19

**Authors:** Michael DeBerardine

**Affiliations:** Department of Molecular Biology and Genetics, Cornell University, Ithaca, NY 14853, United States

## Abstract

**Summary:**

I present here the R/Bioconductor package *BRGenomics*, which provides fast and flexible methods for post-alignment processing and analysis of high-resolution genomics data within an interactive R environment. Utilizing GenomicRanges and other core Bioconductor packages, BRGenomics provides various methods for data importation and processing, read counting and aggregation, spike-in and batch normalization, re-sampling methods for robust ‘metagene’ analyses, and various other functions for cleaning and modifying sequencing and annotation data. Simple yet flexible, the included methods are optimized for handling multiple datasets simultaneously, make extensive use of parallel processing, and support multiple strategies for efficiently storing and quantifying different kinds of data, including whole reads, quantitative single-base data, and run-length encoded coverage information. BRGenomics has been used to analyze ATAC-seq, ChIP-seq/ChIP-exo, PRO-seq/PRO-cap, and RNA-seq data; is built to be unobtrusive and maximally compatible with the Bioconductor ecosystem; is extensively tested; and includes complete documentation, examples, and tutorials.

**Availability and implementation:**

BRGenomics is an R package distributed through Bioconductor (https://bioconductor.org/packages/BRGenomics). Full documentation with examples and tutorials are available online (https://mdeber.github.io).

## 1 Methods and results

### 1.1 Data formatting and quantification

**Figure 1.**
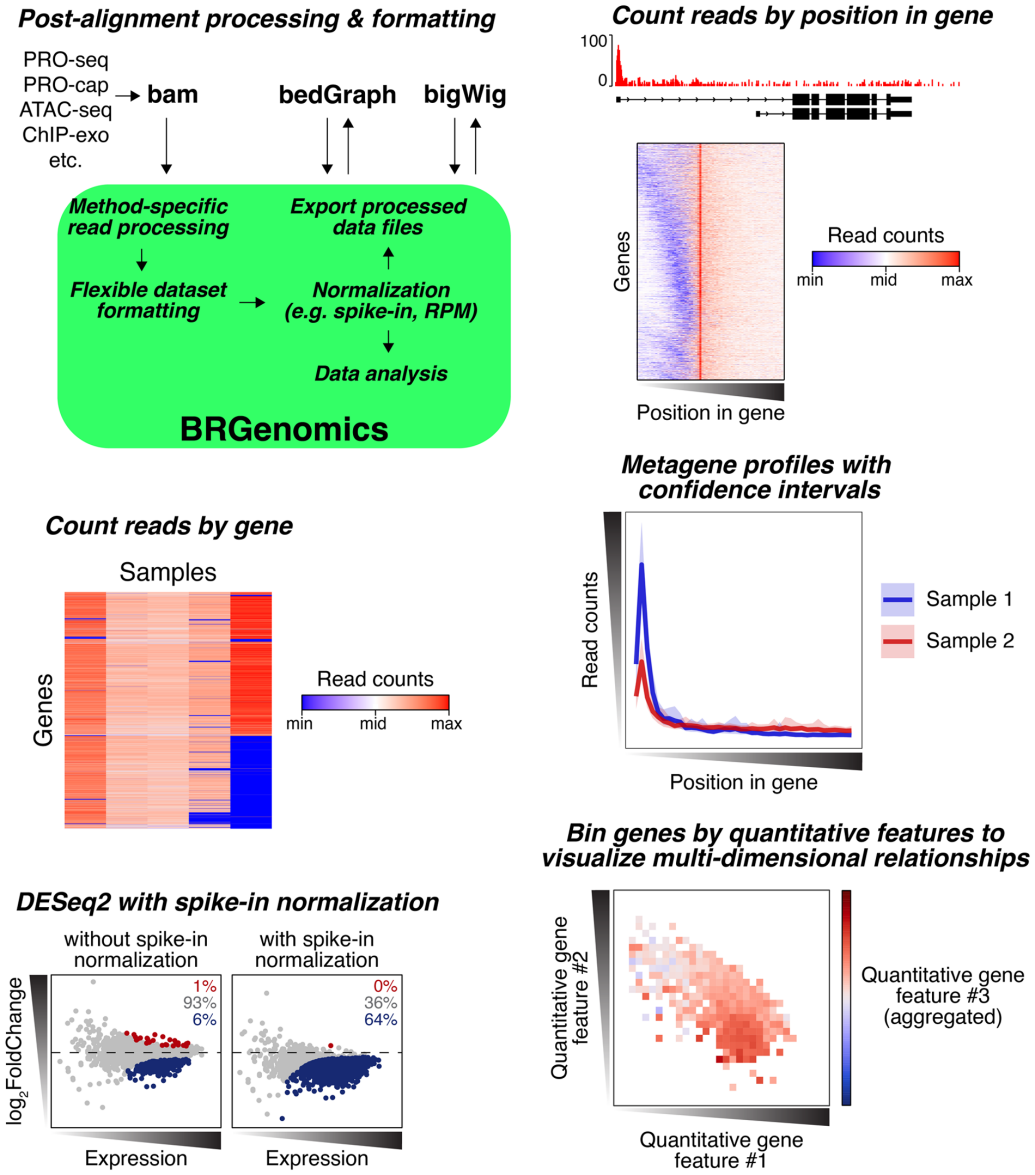
Features and capabilities of BRGenomics.

BRGenomics (‘Basepair Resolution Genomics’) provides straightforward but flexible tools for importing and processing data from various filetypes (bam, bedGraph, bigWig) with various underlying formatting Figure 1. For instance, the function for importing bam files is flexible but comes with some pre-written defaults, including for PRO-seq data (which is reverse complemented and trimmed to the second-to-most 3′ position) and ATAC-seq data (which shifts reads according to their alignment orientation in order to account for the 9 bp space between fragments from a single Tn5 transposition reaction) (Buenrostro et al. 2013; Kwak et al. 2013).

Different kinds of data can be stored and handled differently within R. For instance, a bigWig data track usually employs run-length encoding (RLE), where identical signal values at adjacent positions are merged into a single ‘range’. Data can be kept in this format in BRGenomics, which is efficient for smoothed or windowed data like GC content or whole-read coverage tracks. However, runs of identical signal at adjacent positions are not characteristic of true single-base resolution data, where it’s more efficient to make all ‘ranges’ a single-base span in which each signal count represents a single read. Another approach to data formatting maintains the exact (full) spans of aligned reads, where signal counts represent reads with identical alignments. This is relevant to approaches where paired 3′ and 5′ end information is relevant, such as CoPRO (Tome et al. 2018), but the meaning of the data’s signal counts is entirely distinct from the RLE-style data.

Quantitatively correct handling of all of these data types is accomplished with consistent arguments throughout BRGenomics. Reads can be quantified across entire regions (genes) or at single positions or bins within them, optionally with normalization, blacklisting of certain sites, or different aggregation or resampling methods.

### 1.2 Metagene analyses and profile plots

For ‘metagene’ profiles and related analytical approaches, BRGenomics employs a resampling approach as a robust alternative to means or medians. The default approach samples 10% of the user-supplied annotations 1000 times, and calculates the mean signal at each position/bin at each iteration. Because resampled means are normally distributed (unlike the input data), the distribution of these resampled means provides a robust means of producing confidence intervals about the mean. BRGenomics makes it straightforward to generate profile plots using this approach.

### 1.3 Spike-in normalization

Quantitative genomic methods like RNA-seq, PRO-seq, ChIP-seq, or ATAC-seq can be used to measure global changes between sample conditions if properly normalized. BRGenomics provides methods for filtering spike-in reads (following alignment to a combined genome of experimental and spike-in chromosomes), counting them, and generating normalization factors.

For a given sample, spike-in normalization provides a relative quantification of material obtained. To make these normalized units useful, BRGenomics implements a normalization method in which all samples are put into the same units as an reads per million (RPM) normalized negative control, or spike-in normalized reads per million mapped reads in the negative control (SRPMC), such that the normalization factor for a sample *i* is given by


$${\text{NF}}_{i}=\frac{\sum {\text{Spike Reads}}_{\text{control}}}{\sum {\text{Spike Reads}}_{i}}\cdot \frac{10^{6}}{\sum {\text{Experimental Reads}}_{\text{control}}}.$$

By expressing all counts in the same units as an RPM-normalized unperturbed or wild-type state, SRPMC is maximally portable and interpretable across experiments and studies.

### 1.4 Differential expression analysis with global perturbations

Performing differential expression (DE) analysis when global perturbations are present requires a modified approach to using tools like DESeq2 (Love et al. 2014). BRGenomics uses DESeq2 for DE analysis, but to address global changes, spike-in normalization factors (converted into DESeq2 ‘sizeFactors’) are used, and DESeq2’s ‘blind’ dispersion estimates are avoided. The latter is problematic when some datasets present have global perturbations, as the globally increased dispersion estimates will distort comparisons between any other samples in the dataset. BRGenomics implements wrappers for DESeq2 which enforce strict pairwise comparisons, while also providing a consistent interface as in the other BRGenomics functions.

## 2 Other features and summary

Other features of BRGenomics include support for region blacklisting for all quantifications; methods for binning and aggregating data across an arbitrary number of dimensions; various tools for conveniently modifying annotations (including taking the intersection or union regions of annotated transcripts according to gene annotation); and normalization-by-subsampling approaches. For all details, see the comprehensive user guides and documentation online.
